# *“The one who doesn’t take ART medication has no wealth at all and no purpose on Earth*” – a qualitative assessment of how HIV-positive adults in Uganda understand the health and wealth-related benefits of ART

**DOI:** 10.1186/s12889-022-13461-w

**Published:** 2022-05-27

**Authors:** Uzaib Saya, Sarah MacCarthy, Barbara Mukasa, Peter Wabukala, Lillian Lunkuse, Zachary Wagner, Sebastian Linnemayr

**Affiliations:** 1 Pardee RAND Graduate School, Santa Monica, CA 90401 USA; 2 RAND Corporation, Santa Monica, CA 90401 USA; 3 Department of Health Behavior, University of Alabama at Birmingham School of Public Health, 227, Ryals Public Health Building, 1665 University Boulevard, Birmingham, AL 35233 USA; 4 Mildmay Uganda, Box 24985, Kampala, Uganda

**Keywords:** HIV/AIDS, Antiretroviral therapy (ART) adherence, Uganda, Long-term benefits, Semi-structured interviews

## Abstract

**Background:**

Increases in life expectancy from antiretroviral therapy (ART) may influence future health and wealth among people living with HIV (PLWH). What remains unknown is how PLWH in care perceive the benefits of ART adherence, particularly in terms of improving health and wealth in the short and long-term at the individual, household, and structural levels. Understanding future-oriented attitudes towards ART may help policymakers tailor care and treatment programs with both short and long-term-term health benefits in mind, to improve HIV-related outcomes for PLWH.

**Methods:**

In this qualitative study, we conducted semi-structured interviews among a subsample of 40 PLWH in care at a clinic in Uganda participating in a randomized clinical trial for treatment adherence in Uganda (clinicaltrials.gov: NCT03494777). Interviews were transcribed verbatim and translated from Luganda into English. Two co-authors independently reviewed transcripts, developed a detailed codebook, achieved 93% agreement on double-coded interviews, and analyzed data using inductive and deductive content analysis. Applying the social-ecological framework at the individual, household, and structural levels, we examined how PLWH perceived health and wealth-related benefits to ART.

**Results:**

Our findings revealed several benefits of ART expressed by PLWH, going beyond the short-term health benefits to also include long-term economic benefits. Such benefits largely focused on the ability of PLWH to live longer and be physically and mentally healthy, while also fulfilling responsibilities at the individual level pertaining to *themselves* (especially in terms of positive long-term habits and motivation to work harder), at the household level pertaining to *others* (such as improved relations with family and friends), and at the structural level pertaining to *society* (in terms of reduced stigma, increased comfort in disclosure, and higher levels of civic responsibility).

**Conclusions:**

PLWH consider short and long-term health benefits of ART. Programming designed to shape ART uptake and increase adherence should emphasize the broader benefits of ART at various levels. Having such benefits directly integrated into the design of clinic-based HIV interventions can be useful especially for PLWH who face competing interests to increase medication adherence. These benefits can ultimately help providers and policymakers better understand PLWH’s decision-making as it relates to improving ART-related outcomes.

**Supplementary Information:**

The online version contains supplementary material available at 10.1186/s12889-022-13461-w.

## Introduction

Globally, almost 25.4 million people are now accessing lifesaving antiretroviral therapy (ART) [1]. Increased access to ART has been shown to improve the health, quality of life, and life expectancy of people living with HIV (PLWH) [2–4]. However, maximizing the benefits from ART—such as delayed HIV-related symptoms—depends on optimal retention in care and continued adherence to treatment over time (also called ART persistence). Poor adherence, such as missing doses, could increase a person’s viral load and the risk for transmitting HIV. Data from clinic-based adult populations in sub-Saharan Africa suggests than 21–44% of ART clients have poor adherence [5, 6]. In Uganda, less than half of clinic-based adult PLWH on ART achieve 85% adherence to their ART medication [7], even though 93% of eligible adults receive ART and 82% of PLWH have suppressed viral loads [8]. Few studies have examined adherence among those clinic-based adult PLWH who have been on treatment over the long term [9]. Sustained engagement in HIV care and adherence to ART is largely determined by long-term individual behavior, as well as issues at the household and structural levels (especially those influenced by economic, institutional, political factors) [10–14].

Evidence from the HIV and public health literatures indicate that there are various demand and supply-related reasons for ART initiation and continued adherence—these include socio-demographic and socio-economic characteristics, existing health status, affective factors (such as fear of stigma, depression), social support, as well as institutional and health system barriers [15]. While these factors help understand how ART adherence is shaped, it is equally important to understand how long-term factors can be leveraged to promote ART investment and sustain present-day ART adherence. One way to do this is to consider how such treatment provides benefits in the future. Evidence from the economics literature shows that declining mortality and increased life expectancy shape future-oriented behavior and affect economic choices and human capital investments [16, 17]. The availability of an HIV treatment that increases life expectancy by an average of 18 years [18] could potentially alter how individuals consider health-related risk-taking and information-seeking, and wealth-related investment decisions. Highlighting the perceived benefits of ART adherence in the short and long-term, especially how it affects both health and wealth can be a promising approach to expand ART use.

Prior literature has extensively reported on how existing attitudes and perceptions of ART determine adherence among PLWH [19–22]. In addition, structural, and institutional barriers such as lack of privacy and confidentiality, maltreatment by healthcare workers, and stigma-related factors influence health behaviors of PLWH as well. ART availability may also influence long-term behavior to improve clinical outcomes. HIV literature in this space does not explicitly discuss the role of continued ART adherence in improving these outcomes, nor does it explicitly discuss the economic benefits of being physically healthy after taking ART, but these studies provide useful context to better understand how PLWH perceive benefits in this space and may change their behavior across the HIV care continuum. For example, when ART was made available in Kenya, female PLWH reported a 70% increase in pregnancies and 35% increase in self-reported sexual behavior [23]. These estimates suggest that availability of ART treatment can change health behaviors, but exactly how and why individuals change their behaviors is not well understood. A study from Malawi found that improved ART availability decreased individuals’ self-reported mortality risk as measured by their life expectancy, but also increased labor supply, and future-oriented expenses in their children in the form of their education and clothing expenses [24, 25]. There is also some evidence from South Africa suggesting that increased knowledge about ART-related life expectancy gains had an effect on human capital investments [26].

In terms of the kinds of other broader benefits that ART confers especially on long-term factors such as wealth, increased access may also offer some financial benefits to households by reducing out of pocket spending on medical care [27] or allow for continued employment and higher earnings [28, 29]. These studies demonstrate this increase in wealth by focusing on how ART reduces a variety of health care and non-health care costs (such as burial expenses or opportunity cost of time attending funerals) or reduces anxiety and stress, which may in-turn increase household income. However, despite this strand of evidence, what remains unexplored are the broader benefits of ART reported by PLWH, especially in the qualitative literature. Investigating such factors may help understand short and long-term benefits of ART. Understanding how individuals perceive benefits of ART use on other domains such as wealth can help promote expansion of, and investment in ART.

In this qualitative study, we explored the perceived benefits of ART adherence on health and wealth at the individual, household, and structural levels. We interviewed adult PLWH at an HIV clinic in Kampala, Uganda where the waiting area had visible reminders and cues about the positive influence of ART (Appendix B)—these cues initially served as motivation for exploring our research question more rigorously using one-on-one interviews with PLWH in care. The results from this study can guide policymakers and researchers alike—they can be especially useful since broadening the focus of HIV care and treatment programs to not just the short-term health benefits but also the long-term effects on other domains can be helpful to tailor interventions and improve HIV-related outcomes.

## Methods

Our qualitative study is informed by the Consolidated Criteria for Reporting Qualitative research (COREQ) [30] (Appendix A).

### Study sample

Between July–August 2018, we conducted semi-structured interviews with a purposive sample of 40 the HIV-positive adults who were enrolled in a randomized controlled trial (RCT) called “Behavioral Economics Incentives to Support HIV Treatment Adherence” (BEST) (clinicaltrials.gov: NCT03494777) [31]. This two-year trial is testing the efficacy of using small lottery incentives to support ART adherence for treatment-mature PLWH in care, eventually enrolling 320 participants.

The participants in these semi-structured interviews (as those for BEST) were all patients at Mildmay Uganda, a clinic in Kampala, Uganda that has a longstanding research collaboration with local partners (as well as the BEST study team). Mildmay is a non-governmental organization in Kampala, Uganda that specializes in the provision of free comprehensive HIV/AIDS prevention, care, and treatment services through outpatient and inpatient care for over 15,000 patients. They were recruited into the RCT and were all 18 years of age or older, receiving ART at the participating clinic for 2 or more years, and had demonstrated recent adherence problems within six months of being recruited based on clinical records (defined as showing lack of viral suppression, being sent to adherence counseling, or at disease stage 3 or 4 as per WHO guidelines). Individuals were excluded if they were not mentally fit enough to provide informed consent, spoke neither English nor Luganda (the local language), were participating in any other adherence-related study, were inconsistently using the trial-issued Medication Event Monitoring System (MEMS) cap to monitor adherence. The target sample size for this study was 40 PLWH in care as this was sufficient to achieve saturation, and these individuals enrolled in the parent RCT prior to the baseline survey (and before treatment assignment in BEST). The qualitative sample was chosen via a convenience sample of 40 PLWH in care who enrolled in BEST, and none of the participants who had enrolled in BEST declined to participate in the qualitative study. Specifically, when reviewing the initial round of transcripts, we noticed similar emerging themes cited by participants across the short and long-term benefits of ART. These themes did not improve the explanation of existing themes or add any new ones. This practice follows recommendations from the literature which suggest operationalizing saturation and devising stopping criteria for the number of interviews in which no new themes are identified [32]. Additionally, some literature cites this as a range of 25–30 in-depth, semi-structured interviews [33].

### Recruitment

We used Mildmay’s electronic medical record system to identify eligible participants. Once identified in the clinic database, the study team took note of the subsequent recruitment opportunity based on their next available date of appointment. The study coordinators then looked out for individuals due for a visit that day and approached all eligible participants in-person and inquired whether s/he was interested in participating in an ongoing study. Respondents were assured they would not lose their spot in the queue for any clinic services. Those individuals that initially agreed were taken to a separate study room to verify eligibility, and the survey objective and procedures were explained. Once the participant gave written informed consent to participate in the RCT, s/he was given a MEMS cap to monitor real-time adherence and instructed to store their HIV medication in a pill bottle with the MEMS-cap attached. All participants who had chosen to be enrolled in the RCT agreed to be part of this individual semi-structured qualitative interview. The qualitative interview was then conducted with only the participant and interviewer present in the room, and the participant was compensated USh 20,000 (equivalent to US $5) as a form of transport reimbursement for their time.

### Data collection

A team of one male and one female trained qualitative researchers (co-authors LL and PW) conducted semi-structured interviews in English and/or the local language Luganda at the preference of the participant, with interviews typically lasting 30–40 min. The interviewers (one male, one female) had undergone an extensive 30-h qualitative interview training (led by co-authors US and SM) and had previously conducted other qualitative and quantitative studies with PLWH, especially those with ART adherence challenges. The interview guide (Appendix C) focused on understanding the determinants of ART adherence, as well as the effects of ART adherence on health, life expectancy, and wealth. Prior to interviews with any study participants, the data collection team piloted the interview guide among themselves and other team members to ensure use of appropriate language and local context cues. Open-ended questions focused on the key facilitators and challenges in taking ART, how ART affected health currently and in the future, and whether taking ART as prescribed would also influence wealth and life expectancy.

All interviews were audio-recorded, transcribed verbatim and translated from Luganda into English, and stored on a secure data portal. To ensure confidentiality, we separated personal identification information from the response data, and respondents were only identified through their clinic ID. Approximately 20% of the participant interviews were re-transcribed by an additional team member for quality control and re-evaluated against the original transcript to ensure consistency. The transcripts were then entered into Dedoose software. Transcripts were not returned to participants for comment and/or correction.

The interview team met regularly with two co-authors (US and SM) over the course of three weeks to discuss feedback on how participants described the effects of ART adherence on health and wealth. The team also discussed any problems that came up relating to interviewing goals and techniques. Troubleshooting involved improving the style of interviewer probing especially when they encountered issues such as when respondents said there was nothing stopping them from taking medications and did not report any barriers to taking ART. Interviewers were instructed to probe participants further on these points since they were eligible for the parent RCT (and then this this qualitative study) precisely due to their exhibited adherence-related problems (and should in theory report barriers to taking ART which resulted in adherence-related problems in the first place). Another issue raised was that of translating certain words into the local language, Luganda – for example, the words “health” and “lifestyle” are often interchangeable. As a result, when asked about the effects of ART on one’s health, many respondents provided responses focused on their life goals (e.g., job, home, family etc.) rather than discussing immediate health-related impacts.

Demographic and adherence data on 38 of the 40 PLWH in care were obtained from a follow-up baseline interview in October 2018-January 2019. In 2 cases (5% of overall sample), demographic data were not obtained due to non-participation in the follow-up baseline interview. At this stage, these participants had MEMS-recorded adherence that was less than 30%, which made them ineligible to continue in the parent study. Participants were typically eligible for their baseline interview three months after the pre-baseline visit when the qualitative interviews were conducted. These quantitative data collected at the follow-up baseline interview included age, sex, level of education, marital status, WHO HIV infection stage, employment status, MEMS-measured adherence, and whether participant currently had undetectable viral load (to act as a proxy for the biological response to ART). These data were used as participant descriptors and if relevant, to gauge qualitative differences across groups of participants (e.g., by sex, infection stage etc.).

### Theoretical framework

To thematically categorize data in terms of participants’ attitudes and expectations of future outcomes from ART adherence, we relied on health behavior change frameworks that incorporated behavioral learning theory [34]. In the case of our study sample, we sought to understand how PLWH perceived the benefits of ART adherence in the short and long-term, especially how factors at the social ecological levels shaped how they evaluated their own pill-taking behavior, and especially how individual-level factors could be influenced by structural factors too [35]. The social-ecological model has been extensively studied and used to understand how factors with various domains determine health behavior such as ART adherence [36, 37].

In this study, we hoped to better understand the extent to which factors at the individual, household, and structural levels influenced how PLWH perceived the benefits of ART. The individual level identified intrapersonal influences including the experiences and attitudes towards the long-term impacts of ART adherence, while the household level examined interpersonal factors incorporating social dynamics with family and friends; the structural factors included the larger political and cultural context and includes beliefs such as stigma and fatalism, and beliefs about disclosure to family and friends that may influence individuals’ ability to assess the effects of ART on health and wealth.

### Analysis

We used a combination of inductive and deductive content analysis to categorize data based on emergent themes as well as previously structured hypotheses [38, 39]. We repeatedly read the 40 transcripts to become familiar with the data managed in Dedoose, and coded the data based on recurring key issues and themes. We developed a structured coding framework based on a close assessment of all transcripts that included themes as well as content descriptions, inclusion/exclusion criteria, and sample quotes. Additional codes were created based on reading the transcripts. The coders double-coded eight interviews separately to reach a total of 130 excerpts, after which 30 were randomly picked using a random number generator, and each coder blind-coded them. This resulted in 93% agreement, after which one coder (US) continued coding the remaining interviews. These coders met biweekly thereafter to identify any emerging themes and discuss any questions or concerns. Once all coding was completed, one coder (US) read the excerpts per code application and extracted selected quotes per theme, and then reviewed all coded excerpts and wrote a summary of results. We grouped themes at the levels of the social ecological model and examined the effects of ART use on respondents’ health and wealth. As a final step, we extracted quotations to illustrate common themes or responses among PLWH in care. Each quotation was labeled with the individual’s sex and WHO HIV stage.

## Results

### Sample characteristics

Table 1 describes the sample’s demographic and health characteristics using survey and clinic data. The median age of participants was 32 years (interquartile range 20–45 years) and 50% of the participants were male, 68% were employed, and 55% had completed secondary education or more. More men in the sample had completed secondary education (67%) relative to female respondents (45%). The mean monthly income of participants was USD $43.50. Men in the sample had a higher monthly mean income at $47 compared to their female counterparts ($40) even though more female respondents (75%) reported being employed (largely driven by self-employment) than their male counterparts (61%). Most participants (69%) had a Stage 1 HIV classification (CD4 > 350 cells/μL) compared to 13% and 15% with Stage 2 and Stage 3 or 4 classifications (CD4 < 350 cells/μL), respectively. A little less than half of the sample (45%) was virally suppressed (defined as having less than 200 copies/mL) based on the most recent viral load conducted at the clinic prior to the interview.

**Table 1:** Sample characteristics (*n* = 38)

Variable	N (%)
Sex	
Female	20 (50%)
Male	20 (50%)
Employed	
Not currently employed	12 (30%)
Currently employed	26 (65%)
Unknown	2 (5%)
Language	
Luganda	22 (55%)
English	16 (40%)
Unknown	2 (5%)
Age	
18–39 years	26 (65%)
40 + years	14 (35%)
Highest level of education completed ^a^	
None	3 (7.5%)
Primary	14 (35%)
Secondary	16 (40%)
Vocational	3 (7.5%)
University	2 (5%)
Unknown	2 (5%)
Relationship	
Not in relationship	21 (52.5%)
In relationship	17 (42.5%)
Unknown	2 (5%)
Mean monthly income ^b^	$43.50
Virally suppressed at last clinic viral load (< 200 copies/mL)	18 (45%)
WHO HIV Infection Stage	
Stage 1 (with CD4 > 350 cells/μL)	27 (67.5%)
Stage 2 (with CD4 < 350 cells/μL)	5 (12.5%)
Stage 3 or 4 (with CD4 < 200 cells/μL)	6 (15%)
Unknown	2 (5%)

Data for sex and age obtained from clinic database, so *N* = 40; variables with “Unknown” are those obtained from survey data at baseline (which was collected 3 months after the qualitative study as part of routine study data collection)

^a^ Individuals in Uganda typically obtain vocational education after primary or secondary school education as post-primary or post-secondary training, but always prior to any university training

^b^ Income estimation is based on the sample after excluding 2 outliers due to their disproportionate likely due to data entry error. USD estimates calculated based on exchange rate of 1 USD = 3700 Ugandan Shillings in January 2019

### Qualitative interview findings

We identified several factors pertaining to the perceived effects of ART on health and wealth at individual, household, and structural levels that are described in further detail below. Structural influences operate due to the existing individual/intrapersonal and household/intrapersonal relationships. Some structural factors are more removed from individual control than others, and they may still be inter-connected given the nature of the socio-ecological framework and how these factors affect behavior change. Table 2 presents an overview of the themes that influence the benefits of ART in health or wealth per responses from PLWH in care (along with the relative frequency with which they were mentioned by participants in our sample). We do not aim to show the relationships between themes or show which themes are more important to address in a certain intervention, which is the body of other theoretical work that examines how such variables might fit together. In addition to the themes, Table 2 also provides the relative frequency with which they were mentioned by participants in our sample.

**Table 2:** Summary of themes describing multi-level effects of ART on Health and Wealth

Social Ecological Level	ART Benefits *(“the effect of ART on…”)*	Emerging themes	Relative frequency ^b^
Individual (intrapersonal)	Health (lifestyle)^a^	•Increased physical improvements and lowered susceptibility to illnesses •Adoption of positive long-term habits (e.g., improved nutrition and exercise) that help with physical and mental health	****
	Wealth	•Increased financial earnings and accompanying savings from being able to work more regularly •Increased personal motivation to work harder (with monetary and non-monetary benefits)	******
Household (interpersonal)	Health (lifestyle) ^a^	•Increased ability to do routine things (e.g. school, work, raise children) and plan for future •Reduced engagement in risky behaviors (e.g., unprotected sex and substance use) •Improved social support and motivation from peers and providers	******
	Wealth	•Longer lifespan allows for more earning potential and meeting family responsibilities •Improved social ties (leading to more friends and business opportunities)	****
Structural	Health (lifestyle) ^a^	•Disclosure to close friends and family •Lowered stigma due to lack of illness •Improved appreciation for health care providers •Role of fatalism and the inevitability of death	*********
	Wealth	•Increased importance of forward-looking behavior and civic responsibility (e.g., building businesses to help the economy, helping other PLWH)	***

^a^ The words “health” and “lifestyle” are interchangeable in the local context per the data collection team. As a result, when asked about the effects of ART on one’s health, many respondents provided responses focused on their life goals (e.g. job, home, family etc.) rather than discussing immediate health-related impacts. For the purposes of this analysis, we have combined those themes

^b^ Relative frequencies are denoted by: * discussed by < 25% of respondents (or n < 10), ** discussed by 25–50% of respondents (or *n* = 10–20), *** discussed by > 50% of respondents (or n > 20)

### Individual level factors

Respondents described how they perceived of individual-level benefits to their health (via physical improvements to their health and well-being, being less susceptible to disease, and improved long-term personal habits) and wealth (via increased motivation to work and earning more money).

### Health

#### Increased observable physical improvements and lowered susceptibility to illnesses

Respondents were encouraged by the positive ramifications of taking medication, such as having more energy, gaining weight, and feeling stronger overall. One respondent cited feeling stronger after taking medicine, while another noted that without taking ART, she would feel unwell and weak and credited her ART medication to helping her gain energy and be healthy.

“*My situation is now good…right now my body is okay, I am strong and well because when people look at me, they cannot believe that I even take medicine, even my wife.” – Male, Stage 1*

*“I was badly off, used to be so tiny and had rashes, I even asked the doctor that will I ever get the medication and gain energy again. I was like 25 kilos but now am in 70s, I thank so much this hospital” – Female, Stage 2*

Respondents who tended to be at a higher disease stage also described being less susceptible to infection such as flu and other illnesses once they were adhering to their ART. One respondent described how she had not gotten sick from any illness ever since starting her ART and went as far as to say that without ART, she would not be alive.

*“It has helped me a lot because if it’s not for taking this medication then I wouldn’t be alive. I no longer get diseases that disturb me like back then, it’s now 10 years I have never gotten sick of malaria ever since I started taking the medication and I no longer get rashes. It has really helped a lot” –Female, Stage 2*

Some respondents rationalized how ART can help them; they described factors such as a change in antibodies, lowered CD4 count, or higher viral load. A few of them also described how not taking ART would result in “waking the virus” and HIV would no longer be suppressed.

*“It’s possible because those who made this medicine first researched and found out that if a person uses this medicine and it suppresses the virus that came in the body, so if it sleeps and then antibodies continue to increase and do their work. That means a person can live a long life, because now the virus is suppressed so it’s not doing any effect and every time it wakes up the antibodies have the power to fight” – Female, Stage 2*

*“this one (who misses his dose of ART) might die without realizing it. He might just (have) small flu and we hear that he has died but he caused it himself because the doctor tells you have to take the medicine…If he (is) not using it well, the antibodies won't be moving well in other words the body is weak but this one that takes the body is not weak.” – Female, Stage 1*

#### Adoption of positive long-term habits

Few respondents also reported that taking ART regularly allowed them to adopt habits such as improved nutrition and exercise which would help them sustain their own physical and mental health in the long-term. One respondent cited the example of eating on time, improving personal hygiene, and participating in regular exercise, while another also cited how ART resulted in her going to more routine medical appointments. Others touted its benefits to their spiritual well-being (i.e., ART helped them focus on their religion) and mental health (i.e., ART helped them not worry about factors outside their control and gain more confidence in their actions).

*“(when my body is HIV-free), I wake up and do my exercises well. Feeding well, I eat when it is time for me to eat. Also (I improve upon my) personal hygiene and (do) regular exercise” – Male, Stage 1*

*“(I am) eating well, (doing) exercise, (getting) routine checkups, not worrying. Even holding onto the Lord. “(I am) taking care of myself, I eat in time however small the food is. I also try not to overstress and over think a lot except a few things that may be hard to take in.” – Female, Stage 1*

*“It has helped me to gain confidence and accept myself for who I am” – Female, Stage 1*

### Wealth

#### Increased financial earnings and accompanying savings from being able to work more regularly

Respondents described increases in their own financial earnings and accompanying savings from working more regularly and without interruptions. In the short term, one respondent for example described how she was able to now work and earn more money, while another participant described how his financial status was improved since he was able to work.

*“Now if you take well that medication even your financials you will be moving on but if you don’t take it well it disturbs your financial status because you can be unable to work. But if you take it your financials go on well.” – Male, Stage 1*

*“I can now work and make some money to help around at home because if you cannot take medicine you cannot work” – Female, Stage 1*

**Increased personal motivation to work harder (with monetary and non-monetary benefits)**

Respondents also described ramifications such as motivation and personal drive to work harder (and earn more money). One respondent made the direct connection between taking her medication regularly and having energy to work harder and earn money, while another made the connection to living longer and having more time to work for her future.

*“When I take medicine, I stay healthy and get the energy to work harder and get money but when I do not take medicine, I may fall sick” – Female, Stage 1*

*“Of course, when you’re taking medication, you will have to live longer and that means you will have more time to work for that future. The more you take your medicine well, your body is stronger, and you can work harder.”- Female, Stage 1*

One respondent described other forms of non-monetary “wealth” such as being able to survive for her family and provide for her daughter’s education while another respondent described increased motivation to spread religious messages to others.

*“(I) am not rich, but I work and get some money; but the little I get, I thank God. But even this wealth is there because if you got the virus when you had a child of five years and you educate her till when she completes...... isn't that wealth? Yet some time (ago) you would have died because you did not take well the medication (and) now you see that if I (am) certainly here and got checked when the kid was five years, but now she is educated and even gave birth, so that’s wealth also….my wealth is my children-- the fact (is I) am with them and I thank God for that because there is no wealth greater than a child.” – Female, Stage 1*

*“it will help me with more energy to spread the gospel to people. If I don’t take drugs, I will be sick, and on Sundays it will be my day to go and I won’t be able to make it.”- Male, Stage 1*

Another respondent drew this contrast more starkly by describing the futility of not taking one’s medication, comparing the situation to not having any wealth in the short-term at all.

*“The one who doesn’t take the medication has no wealth at all and he has no purpose on earth… The one who doesn't take the medication might be dying soon.” -Female, Stage 1*

#### Household level factors

Respondents made the connection between ART-related benefits to health and wealth at the household level by describing how ART helped them continue to do routine things and plan for their futures, while also deriving benefits from social support and reduced engagement in risky behavior in the process.

### Health

#### Increased ability to do routine things (e.g. school, work, raise children) and plan for future

Individuals said that after taking ART regularly, they could conduct normal activities such as going to work or school, maintaining a job, and even raising a family.

*“Basically at school, mentally I am very fit. Before my medication, I used to be a person that, I don’t know, maybe fear of my condition but now I am okay” –Female, Stage 1*

The ability to plan for families (and have children who are not HIV positive) was a recurring theme, especially among female respondents.

*“It has helped me because I am now happy, because I did not expect to give birth to a child who is negative, but he is now negative. It gives me encouragement.”—Female, Stage 1*

*“This boyfriend I have first of all he wants kids, and there is some counselor who told that if you’re on medication you might not infect your boyfriend who is not infected, so in my future I might get a person who is not infected and I don’t infect him and even our kids might be normal. And even the future of a job is good because you be taking your medication, when your health too, so you can’t be fired at work.” – Female, Stage Unknown*

#### Reduced unprotected sex and substance use

Male respondents also mentioned how taking ART mitigated the negative influence of family and friends. Respondents reported reduced engagement in risky behavior such as unprotected sex and substance abuse. Interestingly, none of the female respondents cited these factors.

*“What might stop me from living long is maybe going back to something like adultery; I used to take alcohol but its good when you are taking medicine. I also used to take cigarettes and I left it when they told me it wasn’t good for the medication, they told me too to eat well and also (be more mindful of) God because I allowed Him to enter my life. I thank God that He has keep live for long.”- Male, Stage 3*

*“You do not have to go out with girls without protection because even if you take medicine you can still get infected with other diseases that can kill you. Sometimes you take medicine and smoke and (use) alcohol, it affects your life. There is also a bad reaction when you take the medication and take alcohol, it is a bad feeling” – Male, Stage 1*

One respondent suggested that reduced engagement in risky behavior translated to savings because he avoided risky activities that he would have otherwise engaged in had he not been taking his medication.

*“It helps me because a lot of boys I live with as for them, he can spend his money and goes for women but as for me I do not usually do that because I am keeping my money because I know my life depends on the medication because the medication does not interact with alcohol.” – Male, Stage 2*

#### Improved social support and motivation from peers and providers

Respondents also cited additional benefits from ART—one respondent described the positive social support she received from her friends while another cited the support from colleagues whom he met at the clinic who encouraged him to continue taking his medication.

*“Even being with people if I see that the thoughts are coming, I go to them instead of locking myself inside in the house. There is a salon. I can sit there for a while and there are tenants whom I chat with and laugh. I also have a tendency of eating well with people around but if they are not around then it becomes difficult, maybe this thing of my eyes which tend to be painful, and I can’t read the bible and that treats me bad. Because I had novels at home, but now I can’t read them.” – Female, Stage 2*

*“I see (similar situation) from colleagues who come here so I tell myself that I am not the only one infected. Some of my friends encourage me to take the medication because they know that this is my life.” – Male, Stage 2*

### Wealth

#### Longer lifespan allows for more earning potential and meeting family responsibilities

Respondents suggested that in the future, their wealth and that of their household would likely be higher as they wouldn’t be sick and could save more money and be financially independent and support their families.

*“Most times I pray to be my own boss- that’s what am fighting for: to get my own business basically I see a bright future because of the long life I will be having (due to ART). My financial status might increase in the future.” – Male, Stage 3*

Female respondents especially highlighted being able to save and carry out their familial responsibilities such as being able to do more for children e.g., paying their school fees, building a home etc. Such findings also speak to broader structural gender dynamics and the role ART plays in influencing these factors.

*“…I take well my medication I can be alive and work for my kids….(and) taking my medication helps not to get sicknesses, and (then) I can (be able to) wake up early and look for something to eat for my kids.. What I know is if (I am) able to work and earn (due to taking ART), I can take care of my kids and also, I can earn since the kids don’t get sick hence, I can have a little to save.” – Female, Stage 1*

*“So far it is very good that these medicines are free, therefore we can save some money and currently I and my husband have started building a house of our own and my first-born child is in senior Five. She’s 17 years.” – Female, Stage 1*

*“It has helped to get energy to be able to work for my kids, being able to prepare for my kids; before I started taking the medication, I used to be weak, and I couldn’t do anything. But ever since I started taking the medication I can work and even be able to pay school fees.” – Female, Stage 1*

#### Improved social ties (leading to more friends and business opportunities)

Some also suggested the benefits of improved social ties because of taking ART regularly—respondents extended this to widening their friends circle and getting lucrative business opportunities.

*“It (taking ART regularly) has helped me to get a lot of things, seeing new things, living up to my youth age, getting new friends like that” – Male, Stage 3*

*“If you’re sick like coughing or you’re down bedridden, nobody will make business with you. So you take the medicine to stay strong, because nobody will make money if they are bedridden” – Male, Stage 1*

### Structural factors

Respondents described structural factors stemming from the local political, institutional, and cultural context that influenced how they perceived ART benefits; these included comfort around disclosure, lowered stigma, the idea of fatalism, improved relationships and trust with health care providers, and general forward-looking behavior such as having a sense of civic responsibility.

### Health

#### Motivates or enables disclosure to close friends and family

Some reported that taking ART and then getting better is a key driver in being able to disclose HIV status to loved ones. One respondent indicated that taking ART regularly and the promise of subsequent “healing” motivated him to disclose his status to his partner. Another respondent described how taking his medication helped with the stigma he faced prior to taking his medication.

*“I have a girlfriend, but she doesn’t even know that am infected. I have a time when I want to tell her, and I have never touched her like having sex but inside I ask myself “what if she gets to know”, so that makes me take medication hurriedly so that they next time they check me I might be healed. My dream is to heal.” – Male, Stage 1*

#### Improved hope and greater aspirations for future and lowered stigma

Other respondents cited additional effects of ART such as renewed hope and aspirations for the future, especially since lack of illness lowered social stigma associated with HIV and being on ART made them realize they could live longer.

*“….even their social life will be easy in (a) community because the community will not stigmatize them as it will be a warm society as they live socially like any other person as they have accepted their status and lived on with it.” – Female, Stage 1*

*“At first, I could not believe (when I was told about my status) because they told me when I was 8 years that I was positive, and I thought I was going to die like my mother. I am now 19 years; around 11 years have passed by. I did not know that I would reach this far, but if I have reached here, then I know I can go further ahead. I thought I was the only one but I met a man and he told me all his kids were like me and he started taking the medication when he was 10 years old, but he is now around 40 years. That gave me courage and strength and I told myself I am not going to die, I have a life ahead of me.” – Male, Stage 1*

Another respondent described how taking his medication helped with the social stigma he faced prior to taking his medication.

*“Let me say like that time when they told me I had to take the medication, I saw that people were going to start laughing at me and I even wanted to kill myself but then they told me to take the medication hence I will be better than the other normal people. So they told me that don’t kill yourself because (you’re) like a normal person now and no one suspects that you’re infected or not.” – Male, Stage 2*

#### Improved appreciation for health care providers

Few respondents described additional effects of ART via their positive experiences at the clinic interacting with staff and health care providers possibly implying improved trust with the health care system. Specifically, they described how taking ART in turn helped them appreciate the staff who counseled them during their illness (and automatically improved their linkage to care).

*“(Before ART), I was badly off, (I) used to be so tiny and had rashes, I even asked the doctor that will I ever get the medication and gain energy again. I was like 25 kilos but now am in the 70s, I thank this hospital so much and I tell every person I see to go here” – Female, Stage 2*

*“Life is not bad- most times when I come, I normally thank the doctors who work on (me) and I see there is some change, (and) I don’t get sick. I am okay, (and there is so much) difference since I came (initially).” – Male, Stage 3*

*“I think mainly it’s the medical workers, and Mildmay as well--, I appreciate the work they have done. I learnt a lot of things, with my wife helping me as well. A lot the doctors told me to take medicine in time usually after I’ve finished eating, and my wife has also encouraged me to take, even though she is not on the medicine.” – Male, Stage 1*

#### Greater fatalism (especially in thinking about inevitability of death)

Respondents also appeared to describe how being on ART minimized (and not enhanced) their sense or fear of death—some of them attributed this to fatalism and the inevitability of death, tying it closely to their religious beliefs.

*“In my own thinking they say we came from God and it’s where we shall go back but for me, I think even AIDS won’t kill me, I will die of something else. There are God’s plans because you can’t say that I won’t die, if it’s about AIDS me I think I live long but I die of something else.”-Female, Stage 2*

*“I think God is the one that makes for us a calendar and everyone has their own calendar.” – Female, Stage 1*

### Wealth

#### Increased importance of forward-looking behavior and civic responsibility

While most respondents did not allude to broader societal factors that described the effect of ART on wealth, few of them discussed how taking ART helped them think about the future and their civic role such as building a business in Uganda and contributing to the welfare of others, especially other PLWH.

*“(After taking ART), first, my life will add on (and enrich) as a result. I will get more strength so that I can work, because for me, my dream is to become a businessman and to help my country Uganda” – Male, Stage 3*

*“When I take my medication, I hope to get a job in the future, to make my own company producing my own things, help infected people like me. So for me to take my medication I know that in the future I have a dream. That means I have to take the medication to fulfill my dream. ” – Male, Stage 1*

## Discussion

We conducted a cross-sectional qualitative study among clinic enrolled Ugandan PLWH and examined the kinds of benefits they experienced in terms of their health and wealth. Our findings outline the contexts PLWH face when thinking about the broad benefits of ART to their lives, especially their attitudes and expectations of future outcomes resulting from ART. In our study, such benefits included those at the individual, household, and structural levels, and largely focused on their ability to live longer and be physically and mentally healthy, while also fulfilling responsibilities pertaining to themselves (in terms of positive long-term habits and motivation to work harder), others (such as improved relations with family and friends), and society (in terms of improved civic responsibility). Even though the study population was entirely made up of PLWH in care who had documented adherence problems (as evidenced by inclusion criteria for the parent RCT), we observed that respondents in this study perceived these various benefits of ART.

At the individual level, respondents described the health-related benefits of ART in terms of physical health improvements and lack of illness, as well as adoption of positive long-term habits over their longer lifespan. Respondents felt they had more control of their health and well-being because of adhering to ART. Wealth-related benefits included increased financial earnings and increased personal motivation to work harder. Prior research has quantitatively examined how increases in life expectancy stemming from ART can encourage forward-looking behavior because individuals are aware of their lowered mortality risk and subsequently make different life decisions [16, 24]. These studies have sought to examine how PLWH respond to taking ART and change their patterns of risk-taking, information-seeking, and monetary investments in children (especially in education and clothing) [23, 24, 40]. The qualitative findings from our study substantiate and contextualize some of these changes reported elsewhere; respondents on ART believe they will have a longer life with HIV and they can see direct benefits to themselves in terms of both their health and personal wealth.

At the household level, respondents highlighted benefits of ART on health and wealth, such as increased ability to do routine things, plan for their future and have a higher income potential. Female respondents especially described how ART helped them to better plan for their future and raise children who are not HIV positive, while also earning money to meet the needs of their families. This finding corroborates evidence from other studies describing that adherence to ART changes the context of childbearing for PLWH, specifically since having children indicates that PLWH are leading a healthy adult life [41]. A meta-analysis found that the prevalence for fertility desires was 42% among PLWH, and this was higher among male PLWH [42]. Our qualitative findings in this clinic-enrolled sample of PLWH show that females were more likely to describe these ART-related benefits. This could indicate that counseling and reproductive health care be provided to female PLWH who generally would feel a greater sense of responsibility to their families. Male respondents on the other hand described the importance of ART in helping to reduce risky behavior such as unprotected sex and substance abuse. Such behaviors are well established risk factors for poor HIV care outcomes, especially in terms of facilitating testing uptake, engagement and retention in care, and ART use and adherence [43]. Evidence from Kenya and Uganda has shown that alcohol use especially among men was associated with decreased ART use, mostly due to poor engagement and retention in care [44]. Our qualitative findings among male respondents suggest that future ART support interventions may benefit from being more tailored to those with low or medium level alcohol use. As care models of ART delivery branch out of the clinic, adherence support interventions should take advantage of such tailored messaging to incentivize PLWH who could possibly benefit from changes to their benefits and enhance the benefits they derive from ART.

At the structural level, many respondents described the link of ART with comfort in HIV status disclosure, lowered stigma, and increased civic responsibility, as well as increase in appreciation for health care workers at the clinic. Our qualitative findings are in line with related evidence which describes how ART can reduce internalized stigma in Uganda and even hasten disclosure due to improved coping mechanisms and social support [45]. All respondents highlighted the link with employment and ability to increase their earnings if they continued taking their ART. Some of these findings are consistent with studies highlighting the important role of employment in ART adherence. This literature describes the supportive role of employment among PLWH because it is associated with improved adherence; these studies normally describe the factors that determine adherence since financial constraints are a key barrier to ART adherence. A systematic review of ART adherence and employment status described how employed PLWH were 27% more likely to adhere to ART than their unemployed counterparts [7, 46]. Our findings corroborate other studies also reporting that benefits described by our study respondents (e.g. improved wealth, employment etc.) are associated with movement along the HIV care continuum [47, 48]. Respondents who cite these benefits will be more likely to engage in continued care and improved adherence.

One major implication of our findings is that despite knowing about the broad short and long-term benefits of ART, PLWH in care who were part of this study still had challenges in terms of adherence. Literature has discussed various reasons for this, especially among those who have been on ART for several years. PLWH in care may fail to take their pills regularly due to treatment fatigue and waning motivation from engaging in routine pill-taking behavior [49, 50]. Behavioral biases such as present bias—where individuals give in to current temptations and forego future benefits—contribute to suboptimal behavioral such as lowered adherence rates [51–53]. Additional reasons may include not having sufficient education and counseling on ART and possibly not having a lifestyle suitable to continuous pill taking (e.g. living far away from ART refill clinics, experiencing stigma etc.) [54]. Our study findings underscore those from elsewhere that describe how PLWH taking ART for long periods of time fall in and out of adherence. In the case of our study population, they do so despite knowing about the various benefits of ART.

Our study was subject to several limitations; while there were some observable differences in themes across male and female respondents, we were not able to assess if benefits varied based on access, availability, or quality of education, jobs, or other factors related to the benefits described by respondents. While the sample size was large enough to reach saturation, this difference especially between sub-groups may be possible to assess with a larger qualitative sample. Our findings may also differ with a study sample that was not enrolled in a study seeking to improve adherence among low adherers—these individuals may already believe that ART improves health. The ordering of the questions (starting with health, describing effects on life expectancy, and then finally wealth) may have followed a natural order, but perhaps responses would be slightly different if the order were switched. Each participant was also interviewed only once, and had they been interviewed at the end of the 2-year parent study, we could assess if their perspectives had changed, and collected data on whether they experienced first-hand any of the benefits they described. While not exactly a limitation, the study findings point to the discrepancy between participants perceiving the benefits of ART to their health and wealth, yet not adhering to their medication (which is why they are enrolled in a treatment adherence RCT). This could be due to a desirability bias where they may not internalize the messages about ART, or they may be subject to short-term structural and behavioral barriers (such as behavioral biases) that prevent them from fully realizing the benefits they mentioned in the qualitative findings.

Strengths of this study include the fact that we included individuals on long-term ART with recent adherence problems in a clinical setting and investigated the benefits of ART in two distinct domains within their lives: health and wealth. This subject has previously not been studied extensively in literature using qualitative methods and can help understand how PLWH perceive these benefits.

## Conclusions

When recounting the contributions of ART in their lives, PLWH do not simply consider the short-term health benefits, but also the longer-term implications on other domains. This is evident from the findings of this qualitative study. Our study findings can be useful as testimonials for promoting ART expansion, as well as for future programming meant to target these areas as they can help understand how or why PLWH in care perceive certain benefits, especially those that are accrued in the long-term. This is especially germane for those PLWH in care who face adherence problems such as our study population. Our study findings can be used to better promote the expansion of ART and help donors understand the broader benefits of ART (especially the non-health related factors for PLWH in care). We provide contextualized evidence on broader social and economic evidence pertaining to ART. As described by participants, improvements in health via ART can affect future health and income by making individuals more productive (e.g., being able to attend school and get the requisite skills, attain better social networks etc.). Interventions can be designed with these benefits in mind for individuals who don’t specifically recognize such benefits (e.g., treatment initiators who may benefit from knowing about additional reasons to continue to take their medication). Such interventions can specifically use short and long-term individual-level factors to drive improvements in uptake (e.g., by focusing on how at the individual and household levels, ART helps PLWH encourage healthier habits, or helps to increase income, which can ultimately benefit their families).

Findings at the household and structural levels can be useful to clinic staff such as providers or counselors who are encouraging PLWH to sustain ART use. Evidence from other parts of SSA such as in South Africa suggests the need to incorporate this kind of evidence for treatment adherence—in that context, individuals discontinued ART because they were uncertain about its value and overall benefit in their lives [55]. Having benefits that focus on structural factors – such as counseling for their future or resources on ways to get involved helping their communities – directly integrated into the design of interventions can be useful especially in a context when PLWH face so many competing interests to increase medication adherence. These benefits can ultimately help providers and policymakers improve ART-related outcomes. Further research is warranted to determine how these benefits differ across groups of PLWH, especially those with varying levels of access to ART, retention, or linkage to care in HIV care programs, or other sociodemographic factors. Additional research can also examine whether these ART benefits can result in changes in behavior such as increased human capital investments.

## Supplementary Information

**Additional file 1:** **Appendix A.** Consolidated criteria for reporting qualitativestudies (COREQ): 32-item checklist. **Appendix B.** Posters at MildmayUganda. **Appendix C.** Semi-StructuredInterview Guide: Adherence, health, and wealth. 

## Data Availability

Datasets generated and/or analyzed during the current study are not publicly available due the restrictions statement in our study consent forms. Data requests may be routed to Daniel Chung at dchung@rand.org.
