# Supplementary material for: “The one who doesn’t take ART medication has no wealth at all and no purpose on Earth” – a qualitative assessment of how HIV-positive adults in Uganda understand the health and wealth-related benefits of ART
Source: BMC Public Health. 2022 May 27;22:1056. doi: 10.1186/s12889-022-13461-w (PMC9137215; doi:10.1186/s12889-022-13461-w)
Supplement: Supplementary file 1 — Additional file 1: Appendix A. Consolidated criteria for reporting qualitativestudies (COREQ): 32-item checklist. Appendix B. Posters at MildmayUganda. Appendix C. Semi-StructuredInterview Guide: Adherence, health, and wealth. [file 12889_2022_13461_MOESM1_ESM.docx]

***“The one who doesn’t take ART medication has no wealth at all and no purpose on Earth*”** **– a qualitative assessment of how HIV-positive adults in Uganda understand the health and wealth-related benefits of ART**

Corresponding Author: Uzaib Saya

## Appendix

### Appendix A: Consolidated criteria for reporting qualitative studies (COREQ): 32-item checklist

Developed from: Tong A, Sainsbury P, Craig J. Consolidated criteria for reporting qualitative research (COREQ): a 32-item checklist for interviews and focus groups. *International Journal for Quality in Health Care*. 2007. Volume 19, Number 6: pp. 349 – 357

| **No. Item** | **Guide questions/description** | **Reported on Page #** |
| --- | --- | --- |
| **Domain 1: Research team and reﬂexivity** |  |  |
| *Personal Characteristics* |  |  |
| 1. Inter viewer/facilitator | Which author/s conducted the interview or focus group? | 7 |
| 2. Credentials | What were the researcher’s credentials? E.g. PhD, MD | 1 |
| 3. Occupation | What was their occupation at the time of the study? | 1 |
| 4. Gender | Was the researcher male or female? | 7 |
| 5. Experience and training | What experience or training did the researcher have? | 7 |
| *Relationship with participants* |  |  |
| 6. Relationship established | Was a relationship established prior to study commencement? | 7 |
| 7. Participant knowledge of the interviewer | What did the participants know about the researcher? e.g. personal goals, reasons for doing the research | 6-7 |
| 8. Interviewer characteristics | What characteristics were reported about the inter viewer/facilitator? e.g. Bias, assumptions, reasons and interests in the research topic | 6-7 |

| **Domain 2: study design** |  |  |
| --- | --- | --- |
| *Theoretical framework* |  |  |
| 9. Methodological orientation and Theory | What methodological orientation was stated to underpin the study? e.g. grounded theory, discourse analysis, ethnography, phenomenology, content analysis | 9-10 |
| *Participant selection* |  |  |
| 10. Sampling | How were participants selected? e.g. purposive, convenience, consecutive, snowball | 6-7 |
| 11. Method of approach | How were participants approached? e.g. face-to-face, telephone, mail, email | 6-7 |
| 12. Sample size | How many participants were in the study? | 6 |
| 13. Non-participation | How many people refused to participate or dropped out? Reasons? | 6-7 |
| *Setting* |  |  |
| 14. Setting of data collection | Where was the data collected? e.g. home, clinic, workplace | 6 |
| 15. Presence of non-participants | Was anyone else present besides the participants and researchers? | 8 |
| 16. Description of sample | What are the important characteristics of the sample? e.g. demographic data, date | 10-11 |
| *Data collection* |  |  |
| 17. Interview guide | Were questions, prompts, guides provided by the authors? Was it pilot tested? | 7-8 |
| 18. Repeat interviews | Were repeat inter views carried out? If yes, how many? | 8 |
| 19. Audio/visual recording | Did the research use audio or visual recording to collect the data? | 8 |
| 20. Field notes | Were ﬁeld notes made during and/or after the interview or focus group? | 8 |
| 21. Duration | What was the duration of the inter views or focus group? | 7 |
| 22. Data saturation | Was data saturation discussed? | 7 |
| 23. Transcripts returned | Were transcripts returned to participants for comment and/or correction? | 8 |
| **Domain 3: analysis and ﬁndings** |  |  |
| *Data analysis* |  |  |
| 24. Number of data coders | How many data coders coded the data? | 9 |
| 25. Description of the coding tree | Did authors provide a description of the coding tree? | NA |
| 26. Derivation of themes | Were themes identiﬁed in advance or derived from the data? | 9-10 |
| 27. Software | What software, if applicable, was used to manage the data? | 10 |
| 28. Participant checking | Did participants provide feedback on the ﬁndings? | NA |
| *Reporting* |  |  |
| 29. Quotations presented | Were participant quotations presented to illustrate the themes/ﬁndings? Was each quotation identiﬁed? e.g. participant number | Results section |
| 30. Data and ﬁndings consistent | Was there consistency between the data presented and the ﬁndings? | Discussion |
| 31. Clarity of major themes | Were major themes clearly presented in the ﬁndings? | 12 |
| 32. Clarity of minor themes | Is there a description of diverse cases or discussion of minor themes? | 12 |

### Appendix B: Posters at Mildmay Uganda

The posters below from Mildmay Uganda show positive messaging around the impact of ART on life and income, clearly demonstrating the importance of taking ART regularly to improve patients’ lives. Such visible reminders and cues around the clinic serve as motivation for exploring our research question more rigorously using one-on-one interviews with ART clients.


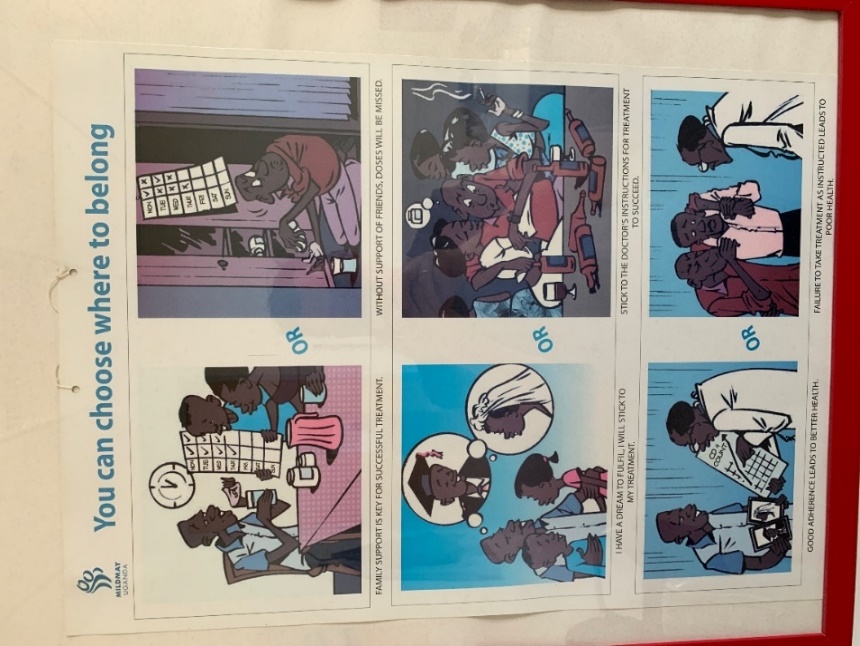

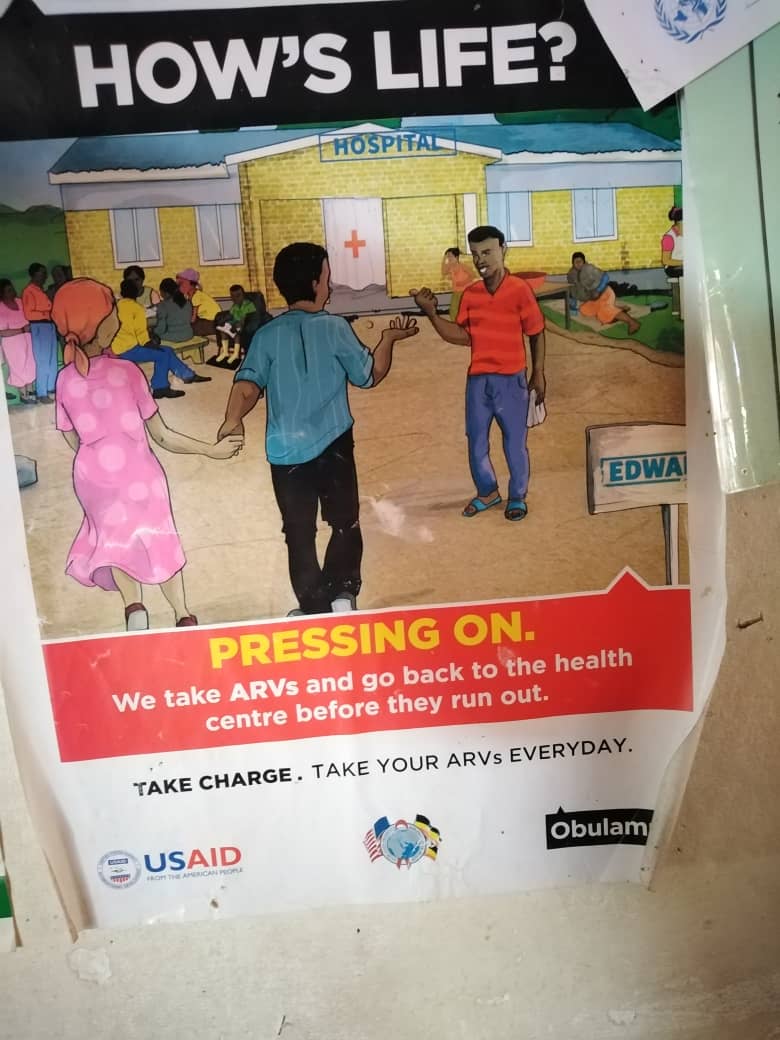


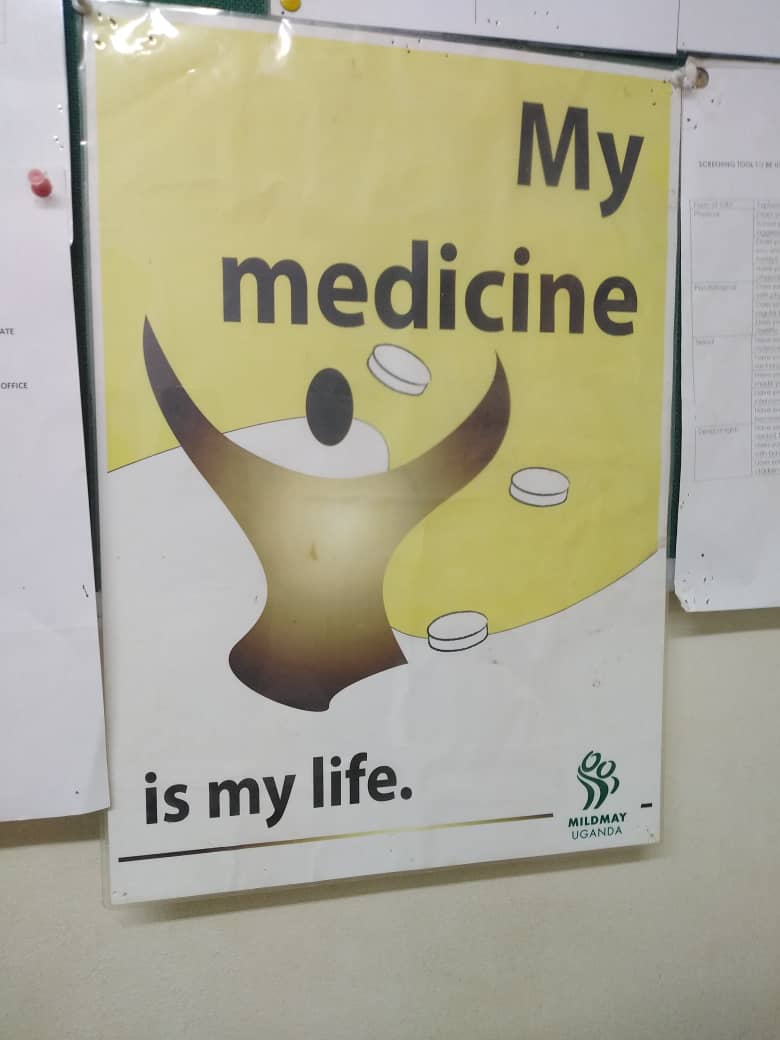


### Appendix C: Semi-Structured Interview Guide: Adherence, health, and wealth

INTERVIEWER INSTRUCTIONS – READ OUT LOUD: I’d like to know a bit more about how people make decisions to take their ART medications and how these decisions influence their wealth. Thank you for talking to me about how you yourself make such decisions. Our talk will last about 30 minutes. I will be recording the conversation. Whatever you say to me will be kept private. Do you have any questions before we begin?

INTERVIEWER INSTRUCTIONS – *Start Recording*

SECTION A: Adherence and Health

**INTERVIEWER INSTRUCTIONS - READ OUT LOUD**: First, I will ask you questions about how you take your ART medications.

1. Please think about taking your ART medications:
   1. What helps you take your ART medications?
   2. What stops you from taking your ART medications?
2. How does taking your ART medications affect your health:
   1. Today?
   2. In the future?
   3. PROBE: *“I know you may face some unique challenges in taking your medications. Could you please help me understand what challenges you face doing this given that you know the medications are important for you?”*

**INTERVIEWER INSTRUCTIONS –** For question 3 below, begin to explain this question by drawing these options on a piece of paper (draw the number 1 and explain this is the person who takes their ART on a regular basis, and draw the number 2 to denote the other who does not take their ART on a regular basis)

1. Imagine there are two different people – one who takes their ART on a regular basis (meaning they never miss a pill) and the other who does not take their ART on a regular basis. How would their health be different?
2. Do you think someone would live longer if they regularly took their ART medications? (Circle: Yes / No?) Why?
3. Do you think someone would live longer if they did not regularly take their ART medications? (Circle: Yes / No?) Why?
4. Other than taking your ART medication:
   1. What helps you live a longer life?
   2. What might prevent you from living a longer life?

**Section B: Adherence and Wealth**

**INTERVIEWER INSTRUCTIONS - READ OUT LOUD:** *We just talked about whether you think ART medications keep you healthy*. *Now, we will talk about how taking your ART affects how much wealth you have, such as how much money you have, or your income.*

1. How does taking your ART medication affect how much wealth you have:
   1. Today?
   2. In the future?
2. If another person was taking their ART medications on a very regular basis (meaning that they never miss a pill)
   1. How would the amount of wealth they have **TODAY** be different?
   2. How would the amount of wealth they t have **IN THE FUTURE** be different?

**INTERVIEWER INSTRUCTIONS –**

**READ OUT LOUD:** *Now, I will lay out some cards with names of some expenses that you may face on a regular basis in your household. I’d like to know more about how you might spend money on these expenses in two different situations (interviewer lays out cards horizontally). Please put these cards in the order of what they would spend the most to least (*note to interviewer: after respondent arranges cards for first column, ask them*, “now, re-arrange if you did not take your ART medications all the time”)*

|  | You always took your ART medications. | You didn't take your ART medications all the time. |
| --- | --- | --- |
| Food |  |  |
| Education/Schooling |  |  |
| Clothing |  |  |
| Medical costs |  |  |

1. Please explain why you changed the order of the cards / kept the order of the cards the same?

*Is there anything else you would like to share about how taking your ART affects how much money you have?*

*Thank you for your time today!*

***INTERVIEWER INSTRUCTIONS –*** *Finish recording; add note if person left early or if there are any issues that we should take into consideration.*
